# 4-Hexylresorcinol Enhances Glut4 Expression and Glucose Homeostasis via AMPK Activation and Histone H3 Acetylation

**DOI:** 10.3390/ijms252212281

**Published:** 2024-11-15

**Authors:** Xiangguo Che, Ji-Hyeon Oh, Yei-Jin Kang, Dae-Won Kim, Seong-Gon Kim, Je-Yong Choi, Umberto Garagiola

**Affiliations:** 1 Department of Biochemistry and Cell Biology, Cell and Matrix Research Institute, School of Medicine, Kyungpook National University, Daegu 41944, Republic of Korea; xiangguo0622@naver.com; 2 Department of Oral and Maxillofacial Surgery, College of Dentistry, Gangneung-Wonju National University, Gangneung 25457, Republic of Korea; oms@gwnu.ac.kr (J.-H.O.); kyj292@hanmail.net (Y.-J.K.); 3 Department of Oral Biochemistry, College of Dentistry, Gangneung-Wonju National University, Gangneung 25457, Republic of Korea; kimdw@gwnu.ac.kr; 4 Maxillofacial and Dental Unit, Biomedical, Surgical and Oral Sciences Department, School of Dentistry, University of Milan, 20122 Milan, Italy; umberto.garagiola@unimi.it

**Keywords:** 4-hexylresorcinol, AMPK, Glut, diabetic complications, histone deacetylase inhibitor

## Abstract

This study investigates the potential of 4-hexylresorcinol (4HR) as a novel antidiabetic agent by assessing its effects on blood glucose levels, Glut4 expression, AMPK phosphorylation, and Histone H3 acetylation (Ac-H3) in the liver. In vitro experiments utilized Huh7 and HepG2 cells treated with varying concentrations of 4HR. Glut4, p-AMPK, and Ac-H3 expression levels were quantified via Western blotting. Additionally, GAPDH activity and glucose uptake were evaluated. In vivo experiments employed streptozotocin (STZ)-induced diabetic rats, with or without 4HR treatment, monitoring blood glucose, body weight, and hepatic levels of Glut4, p-AMPK, and Ac-H3. In vitro, 4HR treatment increased GAPDH activity and glucose uptake. Elevated Glut4, p-AMPK, and Ac-H3 levels were observed 8 h after 4HR administration. Inhibition of p-AMPK using compound C reduced 4HR-mediated Glut4 expression. In STZ-induced diabetic rats, 4HR significantly upregulated Glut4, p-AMPK, and Ac-H3 expression in the liver. Periodic 4HR injections mitigated weight loss and lowered blood glucose levels in STZ-injected animals. Histological analysis revealed increased glycogen storage in hepatocytes of the 4HR-treated group. Overall, 4HR enhanced Glut4 expression through upregulation of AMPK activity and histone H3 acetylation in vitro and in vivo, improving hepatic glucose homeostasis and suggesting potential as a candidate for diabetes treatment.

## 1. Introduction

Diabetes mellitus (DM), characterized by persistent hyperglycemia, represents a significant global health challenge. As of 2021, an estimated 529 million adults worldwide were affected by diabetes, with projections anticipating a staggering rise to 1.31 billion by 2050 [1]. While DM is broadly categorized into type I (insulin-dependent) and type II (insulin-independent), its heterogeneity is increasingly recognized [2]. Uncontrolled diabetes gives rise to acute and chronic complications. Acute complications encompass diabetic ketoacidosis, hyperglycemic hyperosmolar state, lactic acidosis, and hypoglycemia, while chronic complications involve microvascular and macrovascular disorders. Microvascular complications manifest as neuropathy, nephropathy, and retinopathy, whereas macrovascular complications include cardiovascular disease, stroke, and peripheral vascular disease. Effective diabetes management is essential for enhancing quality of life and mitigating certain complications, thus reducing the associated socioeconomic burden [3]. However, given the persistence of complications even with optimal glycemic control, there is an urgent need for innovative treatment strategies [4,5].

Streptozotocin (STZ), a selective pancreatic beta-cell toxin, is widely employed to induce type 1 diabetes mellitus (DM) in animal models. In combination with a high-fat diet, STZ is also used to simulate type 2 DM, making it versatile for studying both types of diabetes [6]. By selectively destroying these cells, STZ leads to a significant reduction in serum insulin levels, resulting in hyperglycemia akin to type 1 DM [6,7]. Beyond its impact on insulin, STZ-induced DM involves mitochondrial dysfunction [8]. The generation of reactive oxygen species (ROS) within mitochondria induces oxidative stress, damaging mitochondrial DNA, proteins, and lipids, thereby impairing mitochondrial function [9,10]. Consequently, energy metabolism disruption occurs, particularly in insulin-sensitive tissues like muscle and liver [11]. Mitochondrial dysfunction in pancreatic beta cells and other insulin-sensitive tissues, such as muscle and liver, exacerbates insulin resistance and promotes cellular apoptosis, rendering STZ-injected animals a valuable model for studying type 1 DM pathophysiology and potential therapeutic interventions [6,7,12].

Additionally, AMP-activated protein kinase (AMPK), a pivotal energy sensor and metabolic regulator, exhibits reduced phosphorylation in STZ-induced diabetic models, notably in renal and hepatic tissues, further complicating metabolic dysregulation [13,14]. Histone acetylation changes have been observed predominantly in pancreatic beta cells [15] but may also affect liver and kidney tissues, which express GLUT2 and are directly influenced by STZ exposure [16]. Diminished AMPK activation contributes to impaired glucose uptake and fatty acid oxidation, underpinning the observed hyperglycemia and lipid accumulation in diabetes [17]. Additionally, histone acetylation, an epigenetic modification influencing gene expression, is typically elevated in STZ-induced diabetic animals [18]. Strategies involving histone deacetylase inhibitors hold promise for mitigating DM complications [19]. Increased histone H3 acetylation (Ac-H3) correlates with enhanced glucose uptake in muscle cells [20].

4-Hexylresorcinol (4HR), a natural phenolic compound, exhibits diverse biological activities, including antioxidative, antimicrobial, and chaperone-like functions [21]. As a chaperone, 4HR interacts with proteins, ensuring their proper conformation and stability, thereby preventing protein misfolding and aggregation—a process linked to cellular stress responses [22,23]. Notably, 4HR’s unique ability to penetrate cellular membranes allows it to exert chaperone activity across various cellular compartments, including mitochondria [24]. Additionally, 4HR acts as a histone deacetylase inhibitor, leading to increased levels of Ac-H3 [25]. This epigenetic modification, coupled with its antioxidative properties, positions 4HR as a promising candidate for diabetes therapy [26]. Previous studies have demonstrated that 4HR administration alleviates diabetes-related complications in bone [27], taste buds [28], and testes [29]. 4HR is widely used in cosmetics for its anti-aging, anti-inflammatory, and skin-lightening effects due to strong tyrosinase inhibition [30]. It also serves as an anti-browning agent in food preservation, although its long-term safety requires further study [31]. In antiseptics, 4HR offers moderate antibacterial effects, while in lozenges, it provides pain relief and virucidal properties beneficial for sore throat treatment [32].

Glucose transporter type 4 (Glut4), primarily found in insulin-sensitive tissues such as skeletal muscle, cardiac muscle, and adipose tissue, plays a crucial role in glucose homeostasis [33,34]. Its movement between the plasma membrane and intracellular vesicles is tightly regulated by an insulin-dependent mechanism or the AMPK pathway. Upon insulin signaling, Glut4 translocates from intracellular storage vesicles to the cell surface, facilitating glucose uptake from the bloodstream into the cells [35]. This process is essential for maintaining normal blood glucose levels and providing energy substrates to cells. Dysregulation of Glut4 function can lead to insulin resistance, metabolic reprogramming, inflammation, and even cancer [36]. Interestingly, 4HR administration increases Glut4 expression in the liver [37]. Given that Glut4 expression can be modulated by AMPK phosphorylation and Ac-H3 [38], we hypothesize that 4HR administration may enhance AMPK activation and histone acetylation. To test this hypothesis, we conducted a comprehensive series of in vitro and in vivo experiments, aiming to elucidate the effects of 4HR on blood glucose levels, uncover its underlying mechanisms, and evaluate its potential as a novel antidiabetic agent.

## 2. Results

### 2.1. The Administration of 4HR Increases Glut4 Expression via Ac-H3 and p-AMPK Mediated Pathway

**Figure 1:**
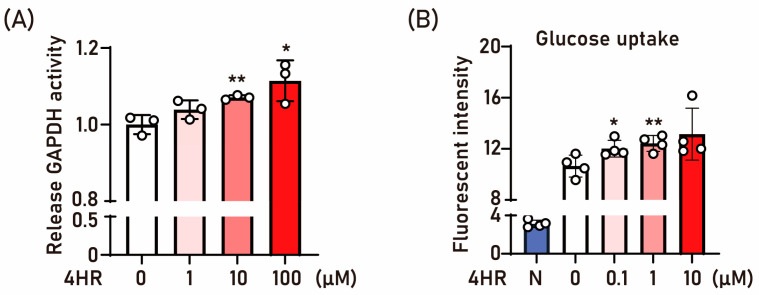
The effect of 4HR on GAPDH activity and glucose uptake. (**A**) GAPDH activity was assessed using an ELISA assay under dose-dependent treatment with 4HR. The administration of 4HR significantly increased GAPDH activity compared to the untreated control. (*n* = 3 for each group, * *p* < 0.05 and ** *p* < 0.01). (**B**) A glucose uptake assay was conducted in HepG2 cells treated with 2-NBDG, a fluorescent tracer, for 2 h in a glucose-free medium, with varying doses of 4HR. The “N” (blue bar) represents the blank condition, with no reagent added (*n* = 4 for each group, * *p* < 0.05 and ** *p* < 0.01).

**Figure 2:**
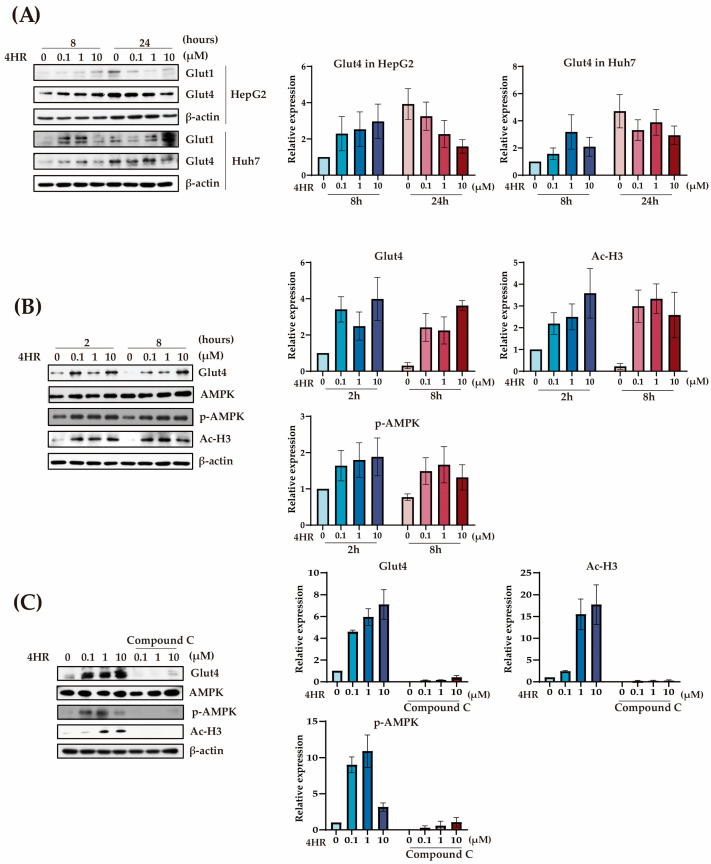
The effect of 4HR on glucose homeostasis-related protein expression in vitro. (**A**) The expression level of Glut1 and Glut4 after administration in HepG2 cells and Huh7 cells. The expression level of Glut4 was increased at 8 h in HepG2 and at 24 h in Huh7 cells after 4HR administration. (**B**) Western blot for Glut4, p-AMPK and Ac-H3 in HepG2. The expression level of p-AMPK and Ac-H3 were increased with Glut4 increase at 2 and 8 h after 4HR administration. (**C**) The application of compound C (the inhibitor for AMPK activation) inhibited the expression of p-AMPK without significant inhibition of AMPK. Consequently, 4HR associated elevation of Glut4 was alleviated by compound C application. Each Western blot experiment was conducted in triplicate.

First, we tested the effect of 4HR on GAPDH because GAPDH is the sixth step of glycolysis and plays an essential in glycolysis as well as in several non-metabolic processes, including transcription activation, apoptosis, ER-to-Golgi vesicle shutting, and axoplasmic transport [39]. The treatment of 4HR increased the GAPDH activity (Figure 1A) and 2-[N-(7-Nitrobenz-2-oxa-1,3-diazol-4-yl)Amino]-2-deoxy-D-glucose (2-NBDG) uptake (Figure 1B).

Next, we checked the expression of Glut4. 4HR increased the expression level of Glut4 at 8 h after treatment in both HepG2 and Huh7 cells (Figure 2A). The activation of AMPK is critical for Glut4 expression [40]. The elevated Ac-H3 is also associated with the increased expression of Glut4 [41]. Indeed, 4HR increased Ac-H3 and p-AMPK at two-time points at 2 h and 8 h. Even 4HR with 0.1 μM concentration increased Ac-H3, p-AMPK, and Glut4 levels (Figure 2B). The compound C has been used as a p-AMPK inhibitor in several experiments [40,42]. When cells were pre-treated with compound C, the expression level of AMPK was not changed significantly. However, the compound C pre-treatment highly suppressed the AMPK activation, as shown in the p-AMPK levels (Figure 2C). Compound C pre-treatment also suppressed 4HR-mediated upregulated expression of Glut4 (Figure 2C). Interestingly, the Ac-H3 level was also highly suppressed by the compound C pre-treatment (Figure 2C).

### 2.2. The Effect of a Single 4HR Injection on Blood Glucose Level

**Figure 3:**
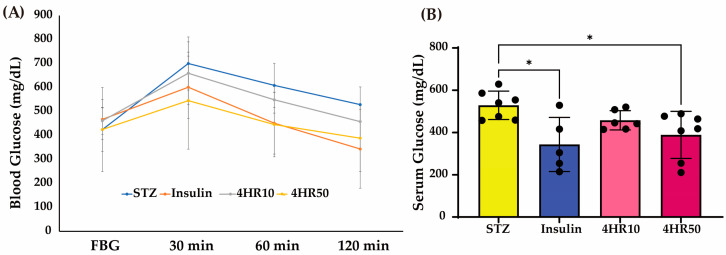
An inhibitory effect of 4HR on STZ-induced diabetes models. (**A**) Glucose tolerance test (FBG: fasting blood glucose). Blood glucose levels increased 30 min after intraperitoneal dextrose injection in all groups. Subsequently, glucose levels decreased until 120 min, with the most prominent decrease observed in the insulin-injected group. Interestingly, the 4HR50 group also demonstrated a comparable decrease in blood glucose levels to that observed in the insulin-injected group. (**B**) Blood glucose levels were measured using a blood glucose assay kit after the administration of insulin, 4HR10, and 4HR50 at 120 min. Multiple comparison tests were performed among groups. Both the Insulin and 4HR50 groups showed significantly lower blood glucose levels compared to the STZ group (*n* = 7 for the STZ group, 5 for the insulin group, 6 for the 4HR10 group, 7 for the 4HR50 group, * *p* < 0.05).

The blood glucose level was increased at 30 min after intraperitoneal dextrose injection in all groups (Figure 3A). Then, the glucose level decreased until 120 min, and the decrease was most prominent in the insulin-injected group (Figure 3A). The blood glucose level of the STZ group was 528.9 ± 66.9 mg/dL at 120 min, and it was 343.7 ± 128.0 mg/dL, 458.0 ± 45.6 mg/dL, and 388.9 ± 111.2 mg/dL for the insulin group, 4HR10 group, and 4HR50 group, respectively (Figure 3B). The difference among groups was statistically significant (*p* = 0.011). In the multiple comparison tests, the difference between the STZ and insulin groups was statistically significant (*p* = 0.012). In addition, the difference between the STZ and 4HR50 groups was statistically significant (*p* = 0.044).

**Figure 4:**
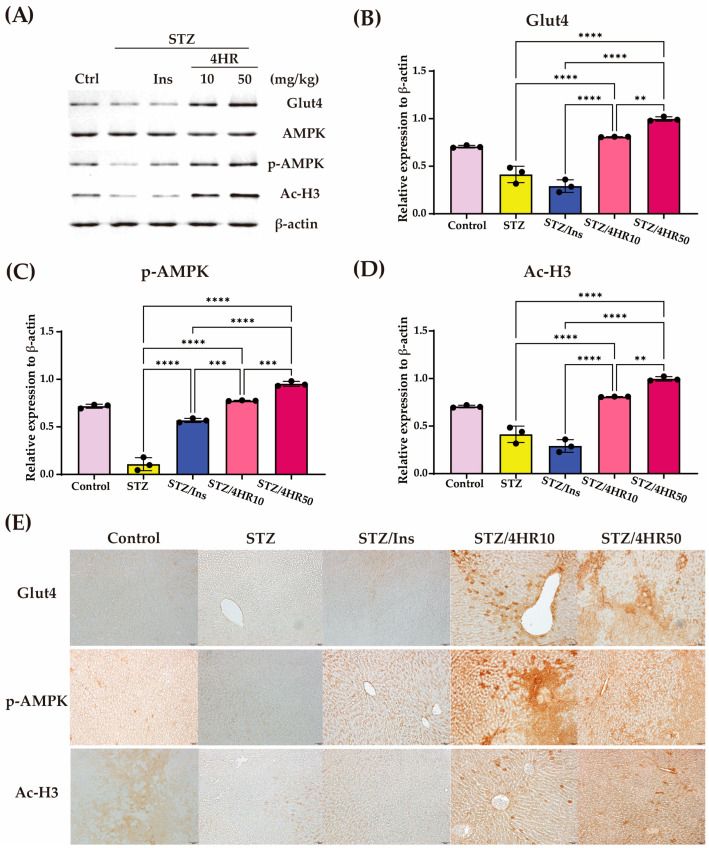
The effect of 4HR on glucose homeostasis-related protein expression in STZ-induced diabetes models. Rats induced with streptozotocin (STZ) were treated with a single injection of either insulin (10 mg/kg), or 4HR at doses of 10 mg/kg (4HR10) or 50 mg/kg (4HR50). Total proteins were extracted from the liver tissues of the rats using RIPA buffer, 2 h post-injection. (**A**) Total protein extracts (10 μg) were used for Western blot analysis. Each Western blot experiment was conducted in triplicate (*n* = 3 for each group). The levels of Glut4 (**B**), p-AMPK (**C**), and Ac-H3 (**D**) were quantified by densitometer (** *p* < 0.01, *** *p* < 0.001 and **** *p* < 0.0001). (**E**) Immunohistochemical analysis of Glut4, p-AMPK, and Ac-H3. Paraffin-embedded slides of each group were stained with each antibody and brown positive signals were shown (original magnification ×200).

Next, we determined the critical markers for glucose metabolism in liver tissue and paraffin-embedded samples (Figure 4). Compared to healthy liver tissue, the STZ group showed decreased activation of AMPK, presenting as the level of p-AMPK and Ac-H3 (Figure 4A). Insulin injection in STZ animals did not significantly increase the Glut4 expression level (Figure 4B). However, insulin treatment increased the activation of AMPK (Figure 4C). Interestingly, 4HR injection increased Glut4, p-AMPK, and Ac-H3 levels compared to the STZ-only group (Figure 4B–D). The immunohistochemical staining of these markers showed a similar increase in expression level as shown in the Western blot analysis (Figure 4E).

### 2.3. The Effect of Regular Drug Injection on Body Weight

**Figure 5:**
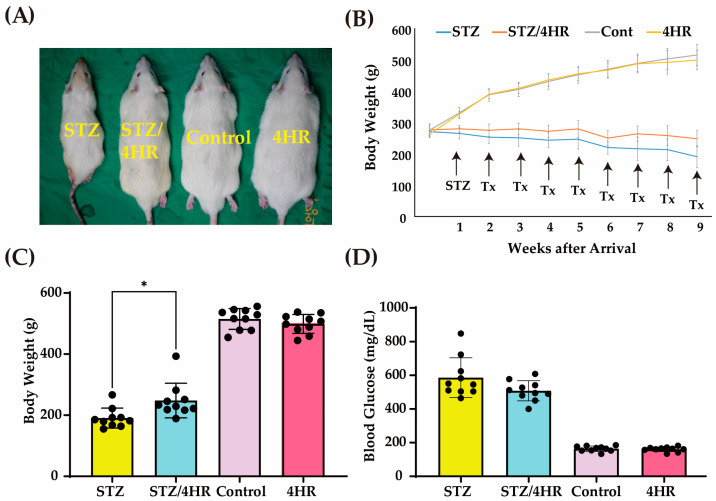
The change in body weight and blood sugar levels under periodic 4HR treatment. Rats in the STZ/4HR group and the 4HR group received 10 mg/kg/week of 4HR for 8 weeks. (**A**) Rat sizes were measured at the time of sacrifice and were taken pictures. (**B**) Rat body weights were measured weekly in each group. The rats that received STZ showed a lower body weight compared to other groups. (**C**) The comparison of body weight among STZ-treated and untreated groups. The STZ/4HR group had significantly higher body weight compared to the STZ group (* *p* < 0.05). (**D**) The comparison of fasting blood glucose (FBG) level. The FBG levels of the STZ and STZ/4HR groups were higher compared to the control and 4HR groups (*n* = 10 for each group).

The rat sizes of the STZ group and STZ/4HR groups were smaller than the control and 4HR groups (Figure 5A). The body weight of the STZ groups showed a continuous decrease after STZ injection, whereas the STZ/4HR group showed less decrease than the STZ group (Figure 5B). Accordingly, the body weight of STZ groups was significantly lower than that of the control and 4HR groups (Figure 5C). The body weight at 9 weeks after animal arrival was 190.7 ± 32.5 g, 248.0 ± 56.6 g, 515.0 ± 34.0 g, and 498.7 ± 31.1 g in the STZ, STZ/4HR, control, and 4HR groups, respectively (Figure 5C). The difference among groups was statistically significant (*p* < 0.001). In the multiple comparison test, the difference between STZ and STZ/4HR groups was statistically significant (*p* = 0.014). In addition, the difference between STZ-injected and STZ-uninjected groups was statistically significant (*p* < 0.001). The fasting blood glucose level at 9 weeks after animal arrival was 585.5 ± 118.3 mg/dL, 507.9 ± 60.2 mg/dL, 164.0 ± 15.9 mg/dL, and 160.5 ± 14.0 mg/dL in the STZ, STZ/4HR, control, and 4HR groups, respectively (Figure 5D). The difference among groups was significant (*p* < 0.001). In the multiple comparison tests, the difference between STZ-injected and STZ-uninjected groups was statistically significant (*p* < 0.001). However, the difference between the STZ and the STZ/4HR groups was not statistically significant (*p* = 0.064).

**Figure 6:**
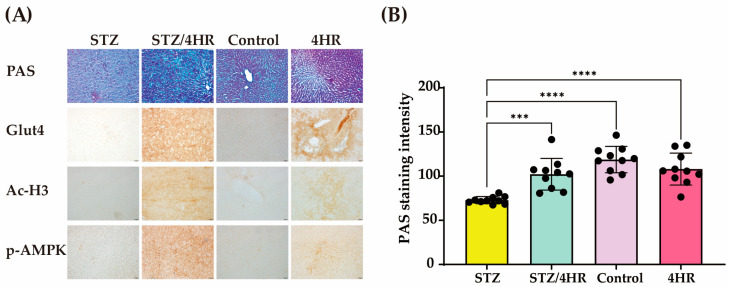
Impaired liver glycogen storage function rescued by periodic 4HR treatment in STZ-induced diabetes models. (**A**) In the case of PAS staining, healthy livers (control and 4HR group) showed a robust magenta color because of the high glycogen content. However, the liver of the STZ-injected group showed weak magenta staining because of significant depletion of glycogen stores. Interestingly, the STZ/4HR group showed intense magenta staining comparable to the healthy liver. The expression level of Glut4, Ac-H3, and p-AMPK were higher in the 4HR and STZ/4HR groups than in 4HR-uninjected groups (original magnification ×200. In the case of PAS staining, counterstained with hematoxylin and light green). (**B**) The quantitative analysis of PAS staining. The PAS staining intensity was significantly lower in the STZ group than in other groups (*n* = 10 for each group, *** *p* < 0.001, **** *p* < 0.0001).

**Figure 7:**
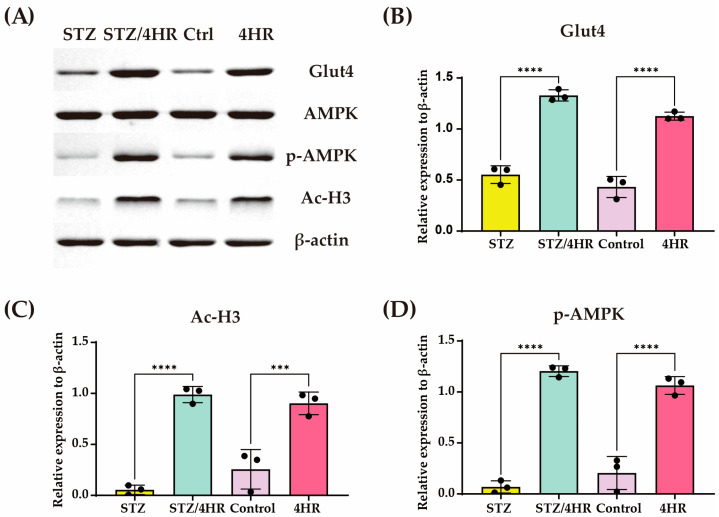
Determination of Glut4, p-AMPK, and Ac-H3 levels of rat liver after periodic injection of 4HR. (**A**) Total protein extracts from rat liver were analyzed using Western blotting to assess the expression levels of Glut4, p-AMPK, and Ac-H3. The expression of these proteins was elevated in the 4HR-treated groups. Quantification of protein levels was performed using specific antibodies for (**B**) Glut4, (**C**) Ac-H3, and (**D**) p-AMPK (*n* = 3 for each group, *** *p* < 0.001, **** *p* < 0.0001). Each Western blot experiment was conducted in triplicate.

In histological analysis, the STZ group showed weak periodic acid–Schiff (PAS)-positive staining, as shown by a patchy appearance of magenta color (Figure 6A). The PAS-positive area was identified outside the hepatocytes, representing increased extracellular matrix components. Both control and 4HR groups showed that the hepatocytes appeared pink to magenta due to the abundant glycogen stored in the cytoplasm. Interestingly, the STZ/4HR group also showed a similar pattern of PAS staining to the control and 4HR group. Glut4, Ac-H3, and p-AMPK were higher in 4HR treated groups (STZ/4HR and 4HR) (Figure 6A). When comparing the intensity of PAS staining among groups, the STZ group showed significantly lower staining intensity compared to STZ/4HR, control, and 4HR groups (*p* = 0.0005 for STZ/4HR and <0.0001 for control and 4HR group) (Figure 6B). Western blot analysis showed these changes after 4HR administration (Figure 7).

## 3. Discussion

The therapeutic potential of 4HR in alleviating STZ-induced complications remains an area of active investigation, particularly regarding blood glucose regulation [27,28,29]. In this study, we explored the mechanistic underpinnings of 4HR’s effects. First, we examined its impact on GAPDH, a key enzyme in glycolysis that also participates in non-metabolic processes such as transcription activation, apoptosis, ER-to-Golgi vesicle shutting, and axoplasmic transport [43]. Given 4HR’s association with dormancy induction in microorganisms [22], we hypothesized that it might modulate energy metabolism, potentially conferring a survival advantage by facilitating cell wall creation and reducing overall metabolism [44]. Notably, protein-ligand interaction simulations revealed 4HR’s high affinity for GAPDH [37]. Consequently, we investigated 4HR’s effect on GAPDH activity directly using recombinant GAPDH, revealing an increase in GAPDH activity following 4HR administration (Figure 1A). Under diabetic conditions, GAPDH activity is decreased [45], which is associated with increased oxidative stress [46]. Given that oxidative stress and cellular apoptosis are associated with diabetes [27,28,29,47] as well as impaired GAPDH activity, this 4HR-mediated increased activity of GAPDH may hold promise for mitigating diabetes-associated complications.

Furthermore, 4HR treatment enhanced cellular glucose uptake (Figure 1B). Glucose transporter proteins, including Glut1 to Glut4, play pivotal roles in intracellular glucose uptake [48]. Among the Gluts, Glut4 is the predominant form in the liver. In our study, 4HR administration increased Glut4 expression in both HepG2 and Huh7 cells (Figure 2A). 4HR concurrently elevated Ac-H3 and p-AMPK levels at 2 and 8 h (Figure 2B). The activation of AMPK is critical for Glut4 expression [49]. The elevated Ac-H3 is also associated with the increased expression of Glut4 [38]. To dissect the mechanisms, we employed compound C, a p-AMPK inhibitor [49]. Pre-treatment with compound C effectively suppressed AMPK activation without changing its expression level and attenuated 4HR-mediated Glut4 expression (Figure 2C). In STZ-induced diabetic rats, Glut4 and p-AMPK expression is reduced [50]. Several antidiabetic compounds, including anthocyanin-rich extract [51], malonyl ginsenoside [52], and shihunine-rich extract [53], exert their effects via the Glut4 and p-AMPK pathway. The observed suppression of Ac-H3 by compound C adds further complexity to this interplay (Figure 2C).

Several models have been developed for cellular experiments simulating glucose intolerance in hepatocytes, such as long-term culture under high glucose concentrations. However, maintaining cellular viability under such harsh conditions is challenging. Moreover, cells exhibiting glucose intolerance should display low levels of GLUT expression in response to glucose administration, but this condition is difficult to replicate in cell culture models. Diabetic animals, on the other hand, inherently have glucose-intolerant livers, making them suitable for testing the effects of drugs. In this context, the diabetic animal model can be considered a living incubator. In our investigation, blood glucose levels were increased 30 min after intraperitoneal dextrose injection across all groups (Figure 3A). Subsequently, glucose levels gradually declined over the next 120 min, with the most pronounced decrease observed in the insulin-injected group (Figure 3A). A single 4HR administration in STZ-induced diabetic rats led to increased expression levels of Glut4, p-AMPK, and Ac-H3 in the liver (Figure 4). Notably, while insulin effectively reduced serum glucose levels as shown in Figure 4, it did not enhance Glut4, p-AMPK, and Ac-H3 expression to the same extent as 4HR (Figure 4). Interestingly, Date-seed extract, known for its non-insulin-dependent effects, also modulates Glut4 and p-AMPK expression [54]. Given that 4HR is also derived from plant extracts [41], its anti-diabetic action may share similarities with other plant-derived compounds.

Since the drug is typically administered periodically, its actual effect on the target disease should be monitored through long-term administration results. The STZ and STZ/4HR groups exhibited smaller rat sizes compared to the control and 4HR groups (Figure 5A). After STZ injection, the body weight of the STZ groups consistently decreased, while the STZ/4HR group showed a less pronounced decline (Figure 5B). Histological examination revealed weak PAS-positive staining in the STZ group, characterized by a patchy appearance (Figure 6A). Chronic diabetic conditions can induce glycogen depletion in the liver [55]. The PAS-positive area, located outside hepatocytes, indicated increased extracellular matrix components in the diabetic condition [56]. In contrast, both the control and 4HR groups exhibited hepatocytes with pink to magenta staining due to abundant cytoplasmic glycogen storage. Interestingly, the STZ/4HR group displayed a PAS staining pattern like the control and 4HR groups. Thus, 4HR administration alleviated glycogen depletion in the diabetic condition.

Histone deacetylase inhibitors (HDACi) have emerged as promising antidiabetic agents due to their ability to modulate inflammation associated with diabetes [57,58]. Among these, 4HR stands out as an HDACi with multifaceted effects [25]. In the context of diabetes, elevated Ac-H3 levels are generally observed in STZ-induced diabetic animals [18], often linked to increased expression of pro-inflammatory cytokines such as tumor necrosis factor-α (TNF-α) and cyclooxygenase-2 (COX-2) [59]. However, 4HR administration counters this trend by suppressing TNF-α expression via inhibition of the nuclear factor-κB (NF-κB) pathway [21]. Additionally, 4HR exhibits potent antioxidant [60] and anti-inflammatory properties [61]. The intricate interplay between HDACs and insulin signaling pathways contributes to glucose homeostasis. For instance, the binding between HDAC2 and insulin receptor substrate-1 (IRS-1) can impair insulin receptor-mediated tyrosine phosphorylation, leading to insulin resistance [62]. Furthermore, myocyte enhancer factor 2 (MEF2), a key transcription factor for Glut4 expression in muscle, is inhibited by HDAC4 and HDAC5 [38]. Remarkably, 4HR administration reduces the expression levels of HDAC4 and HDAC5 [63], potentially mitigating diabetic complications.

In our study, 4HR administration increased Ac-H3 expression in the liver of STZ-injected rats (Figure 4 and Figure 7). This elevation in Ac-H3 was concomitant with increased Glut4 and p-AMPK levels (Figure 4 and Figure 7). The elevated expression of p-AMPK and Ac-H3 is associated with the increased expression of Glut4 [38]. Notably, AMPK activation is crucial for glucose uptake [64] and mitochondrial metabolism [65,66]. Thus, the 4HR-mediated elevation of Ac-H3 and AMPK activation holds promise as a strategy to alleviate diabetic complications.

**Figure 8:**
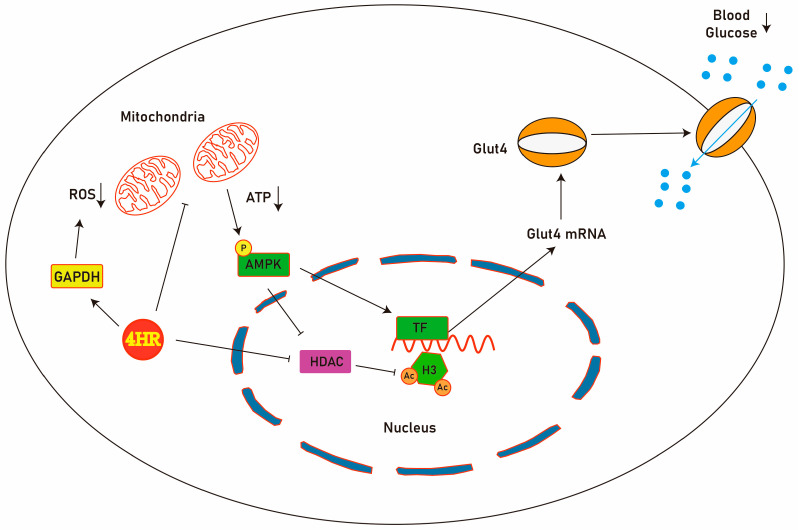
Regulatory mechanism of 4HR on glucose homeostasis. 4HR decreases mitochondria metabolism, which increases AMPK activity. 4HR enhances GAPDH activity with a reduction of reactive oxygen species production and supports mitochondrial survival. Additionally, 4HR functions as a histone deacetylase (HDAC) inhibitor. The activation of AMPK further inhibits HDAC. Furthermore, these actions enhance the transcription of Glut4 and facilitate the intracellular uptake of glucose. Consequently, blood glucose levels can be decreased by 4HR.

4HR administration increased GAPDH activity (Figure 1A), which might reduce the oxidative stress of diabetes (Figure 8). In diabetic animals, reduced levels of p-AMPK are observed [40]. Metformin, a well-known AMPK activator, restores mitochondrial function in diabetic models by indirect activation via inhibition of mitochondrial ATP production [67,68]. Similarly, 4HR inhibits mitochondrial ATP production by preventing succinate formation [24]. The accumulation of succinate can increase mitochondrial ROS through complex I-dependent reverse electron transfer [65]. Decreased succinate by 4HR might lead to acetyl-CoA accumulation, essential for histone acetylation [65]. The activation of AMPK inhibits Class I and II HDAC activity [68]. 4HR acts as a Class I and II HDAC inhibitor [25] but paradoxically increases the expression of Class III HDACs (SIRT3 and SIRT6) [69]. SIRT6, activated by 4HR, counteracts mitochondrial dysfunction by promoting AMPK activity [70]. Collectively, 4HR administration increased Ac-H3 and Glut4 expression (Figure 2, Figure 4, and Figure 7). Enhanced Glut4 likely improves glucose uptake, contributing to reduced blood glucose levels (Figure 8). In summary, 4HR holds promise as a multifaceted therapeutic agent for diabetes management.

The study has several limitations. First, the animal experiment using periodic injections preceded the cellular and single injection models. Weekly 10 mg/kg doses of 4HR did not sustain its effect, failing to reverse the STZ-induced weight loss. Second, the study did not explore AMPK targets like CRY1, CRTC2, HDAC4/5, YAP, FOXO3A, and GLI1 [39], which warrants further investigation. Third, compound C, used as an AMPK inhibitor, also inhibits other kinases [71], limiting its specificity for AMPK functions. Fourth, while 4HR shows potential therapeutic benefits, its use may pose risks of adverse effects or unintended interactions with non-target proteins and pathways, particularly in clinical settings. For example, as a phenolic compound, 4HR may impact metabolic and cellular pathways beyond its intended effects, potentially leading to systemic issues such as irritation of the skin, eyes, and mucous membranes, or more serious complications with prolonged exposure [72]. Additionally, 4HR’s activity as HDACi could broadly influence epigenetic regulation, potentially altering gene expression in non-target tissues. Therefore, thorough safety evaluations and further studies are necessary to fully understand and mitigate potential risks. Finally, animal study results cannot be directly applied to clinical practice; clinical trials are needed to evaluate 4HR’s potential in diabetes treatment.

## 4. Materials and Methods

### 4.1. Antibodies and Reagents

The summary of used antibodies is as follows (Table 1).

The compound C (CAT#: 171261), 2-Deoxy-2-[(7-nitro-2,1,3-benzoxadiazol-4-yl)amino]-D-glucose (2-NBDG) (CAT#: 72987), and insulin (CAT#: I2643) were obtained from Merck (Rahway, NJ, USA). The glyceraldehyde 3-phosphate dehydrogenase activity assay kit was sourced from Abcam (Cambridge, UK).

### 4.2. Cell Cultures

Huh7 human hepatocyte cells (Korean Cell Line Bank No. 60104, Seoul, Republic of Korea), characterized by normal GLUT protein expression, were cultured in RPMI-1640 medium (Hyclone, Logan, Rockland, ME, USA) supplemented with 10% fetal bovine serum (FBS; Gibco-BRL, New York, NY, USA) and 1% penicillin/streptomycin (Lonza, Rockland, ME, USA). Human hepatocarcinoma HepG2 cells (Korean Cell Line Bank No. 88065) were maintained in MEM/EBSS medium (Hyclone, Logan, USA) with 10% FBS and 1% penicillin/streptomycin. Both cell lines were seeded at a density of 2 × 105 cells per well in 6-well plates and treated with varying concentrations of 4HR (0, 0.1, 1, or 10 μM) for specified durations (2, 4, 8, and 24 h). Protein extraction followed the treatments. To evaluate p-AMPK inhibition, concurrent treatment with 10 μM compound C (Sigma-Aldrich, St. Louis, MO, USA) was performed alongside the 4HR administration.

### 4.3. Western Blot Analysis

Protein lysates were obtained after 4HR treatment from cells (Huh7 or HepG2 cells) or rat liver using radioimmunoprecipitation assay (RIPA) buffer. Protein quantification employed a Bradford protein assay kit (Bio-Rad, Hercules, CA, USA). Total proteins (20 µg) were separated on SDS-polyacrylamide gel electrophoresis, and subsequently, proteins were transferred to polyvinylidene difluoride (PVDF) membranes for Western blot analysis. Following blocking with 5% skim milk, the primary antibodies were applied overnight at 4 °C. Enhanced chemiluminescence detection system facilitated protein visualization (GE Healthcare, Uppsala, Sweden).

### 4.4. Glucose Uptake Assay

HepG2 cells, plated at a density of 1 × 10^4^ cells/well in 96-well black plates, reached 90% confluence before 4HR treatment. Cells were exposed to 0, 0.1, 1, and 10 µM 4HR for 2 h prior to glucose uptake analysis. The glucose uptake rate, as previously described, involved adding the 2-NBDG tracer to a glucose-free culture medium [73]. Fluorescence measurements were performed using a FlexStation 3 Multi-Mode Microplate Reader (Molecular Devices, CA, USA) with excitation at 488 nm and emission at 520 nm.

### 4.5. Glyceraldehyde 3-Phosphate Dehydrogenase (GAPDH) Activity Assay

To directly assess 4HR’s impact on GAPDH activity, we employed the GAPDH Activity Assay Kit (Abcam, CAT#: ab204732) following the manufacturer’s instructions. Briefly, recombinant GAPDH (2 µL) and varying 4HR concentrations (0, 0.1, 1, and 10 µM) were adjusted to 50 µL with GAPDH Assay Buffer and loaded into 96-well black plates. Subsequently, the reaction mixture (50 µL) was added to each well, and the kinetic mode measurements were taken at OD450 nm using a Multiskan SkyHigh Microplate reader (Thermo Fisher Scientific, Waltham, MA, USA).

### 4.6. Animal Experiments

The STZ-induced diabetic rat model was selected due to its established ability to induce a diabetic state through selective destruction of pancreatic beta cells, resulting in insulin deficiency and hyperglycemia. This model reliably replicates key aspects of type 1 diabetes, providing a consistent platform for evaluating antidiabetic interventions such as 4HR. Its use allows precise control over the timing and progression of diabetes, facilitating the detailed assessment of metabolic and regulatory markers.

Given the expected metabolic effects of STZ-induced diabetes, significant weight loss often occurs in treated animals. In standard models, a body weight loss of 20% typically warrants euthanasia to ensure humane treatment. However, this threshold was adapted in our study to account for the anticipated weight loss associated with diabetes, ensuring the validity of comparisons with the STZ/4HR-treated group. To balance humane care with scientific objectives, we closely monitored the animals’ overall health, including hydration status, activity levels, and signs of distress, and provided supportive care as necessary to maintain welfare throughout the study.

#### 4.6.1. Single Injection Effect

**Figure 9:**
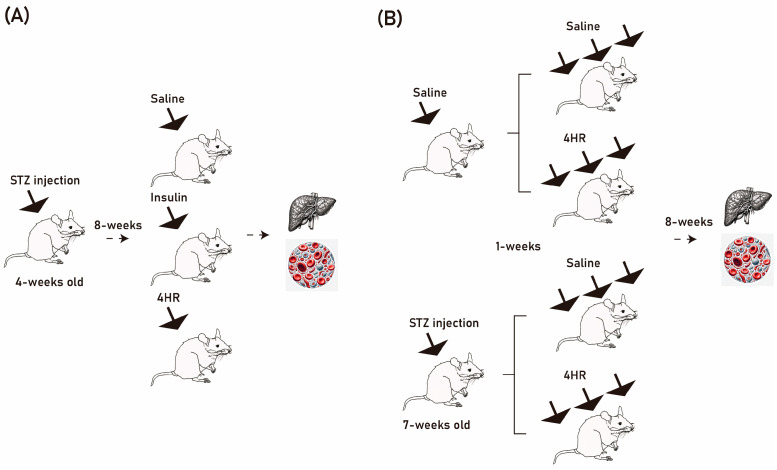
The diagram details the process of 4-Hexylresorcinol treatment in a streptozotocin-induced diabetic animal model. (**A**) A single injection model was used to evaluate the effect of 4HR administration on the expression levels of Glut4, AMPKα1/2, p-AMPKα1/2, and Ac-H3 in a streptozotocin (STZ)-injected animal model. (**B**) A periodic injection model was used to perform additional experiments evaluating the effect of periodic 4HR administration on the body weight and fasting blood glucose levels of both STZ-injected and normal animals.

To investigate the impact of 4HR treatment on Glut4, AMPKα1/2, p-AMPKα1/2, and Ac-H3 expression, we employed an STZ-injected Sprague-Dawley rat model. This study received approval from the Institutional Animal Care and Use Committee at Gangneung-Wonju National University (GWNU-2023-26) on 17 November 2023 (Figure 9A). Thirty male rats, all 4 weeks old and sharing common ancestry, were included. Upon arrival, rats weighed 87–90 g and underwent a 1-week adaptation period. Fasting for 6 to 8 h preceded STZ-induced diabetes via intravenous tail vein injection (40 mg/kg). Rats were subsequently provided regular food and 10% sucrose water.

The following day, blood glucose levels were monitored using the tail vein blood-glucose monitoring system. Animals with blood glucose levels exceeding 300 mg/dL were selected for further study. Those with insufficient levels received additional STZ injections, with at least two injections required for successful DM induction. After 8 weeks post-STZ injection, 28 animals survived until the sacrifice point. Among them, 3 animals exceeded 400 g in body weight and were excluded from analysis. Due to the initial mortality rate and exclusions based on weight criteria, slight variations in the number of animals per group were necessary to maintain adequate sample sizes. Additional animals were allocated to the STZ and 4HR groups, with the Insulin group serving as a positive control based on its expected therapeutic effects on diabetes. These adjustments ensured the reliability of comparisons between groups: STZ group (*n* = 7), Insulin group (*n* = 5), 4HR10 group (*n* = 6), and 4HR50 group (*n* = 7). Prior to the glucose tolerance test, rats fasted for 12 h but had access to water freely. Baseline blood samples measured fasting glucose levels, and body weight was recorded. If the blood glucose exceeded 600 mg/dL, diluted blood was used for measurement.

For the glucose tolerance test, a 50% glucose solution in sterile saline was prepared (standard dose: 1–2 g/kg body weight). Injection volume was adjusted based on the rats’ weight. The glucose solution was administered intraperitoneally using a sterile syringe and needle to minimize stress. The STZ group received normal saline (negative control), while the insulin group received 1 IU/kg of human insulin (Sigma-Aldrich, St. Louis, MO, USA, CAT#: I2643; positive control). In experimental groups, 4HR was administered at two dosages: 4HR10 (10 mg/kg) and 4HR50 (50 mg/kg). Blood glucose levels were assessed 30 min, 1 h, and 2 h post-injection. Subsequently, all animals were euthanized for further analysis.

#### 4.6.2. Effects of Periodic 4HR Administration on Diabetic and Healthy Rat Models

To assess the impact of periodic 4HR administration, we conducted additional experiments using both STZ-induced diabetic rats and normal rats (GWNU-2021-21 and GWNU-2021-22) (Figure 9B). Initially, 40 male rats, all 6 weeks old and sharing common ancestry, were included. Upon arrival, rats weighed 230–260 g, and after a 1-week adaptation period, their body weight ranged from 270 g to 290 g. Twenty rats were allocated to the STZ group. Following a 6- to 8-hour fasting period, STZ was intravenously injected through the tail vein to induce diabetes. Blood glucose levels were measured the next day using a tail vein blood-glucose monitoring system. Animals with blood glucose levels exceeding 300 mg/dL were selected for further study, while those with insufficient levels received additional STZ injections. The STZ-injected animals were randomly assigned to either the untreated control (STZ group) or the 4HR injected group (STZ/4HR group). After confirming the diabetic condition, 4HR (10 mg/kg) was subcutaneously administered weekly throughout the 8-week experiment. Due to the loss of one animal from each group during the study, supplementary animals were recruited (*n* = 1 for each). The remaining 20 rats were assigned to the healthy model and received either the same 4HR dosage as the diabetic model (4HR group) or remained untreated (control group). Body weight changes were periodically monitored. At 16 weeks of age, rats were humanely anesthetized with enflurane and sacrificed. Before sacrifice, fasting blood glucose levels were measured, and whole blood was collected from the heart and stored in heparinized tubes. After centrifugation, serum supernatant was collected for further analysis. Animals were euthanized via paraformaldehyde injection after blood sampling.

### 4.7. Histological Analysis

Paraffin blocks were sectioned into 5 μm thick slices and mounted on glass slides. Dewaxing and rehydration using a series of xylene and decreasing ethanol concentrations removed the paraffin wax from the sections. Hematoxylin staining was performed for 6 min, followed by a brief dip in 1% acid alcohol to remove excess hematoxylin. Subsequently, the slides were immersed in eosin solution for 1 min, staining the cytoplasm and extracellular matrix. After rinsing in tap water, the slides underwent dehydration again using an increasing ethanol series. Cleared with xylene or a xylene substitute, the slides were mounted with a glass coverslip and allowed to dry before examination under a light microscope.

For PAS staining, a commercially available kit (CAT#: ab150680, Abcam, Cambridge, UK) was used to detect glycogen, glycoproteins, and glycolipids in tissues. Following fixation, dehydration, clearing, embedding, sectioning, and deparaffinization, tissue sections were treated with a periodic acid solution to oxidize tissue components for 5 min. After incubation, slides were carefully rinsed with distilled water to remove residual acid and stained with Schiff’s reagent, which reacts with the oxidized tissue components for 15 min. The excess reagent was washed off with lukewarm tap water for 20 min, allowing full-color development. Counterstaining with hematoxylin for 1-min enhanced contrast between different cellular components. The slides were rinsed with tap water or immersed in a bluing solution to stain cell nuclei blue. The tissue sections were dehydrated using an ascending ethanol series to prepare the slides for microscopic examination. Following dehydration, the slides were cleared with xylene or a substitute, allowing the mounting medium and glass coverslip to adhere correctly. Additional copies of slides were stained without counterstaining them for the quantitative analysis. Staining intensity was measured using SigmaScan Pro 5.0 (SPSS Inc., Chicago, IL, USA). In the black and white images, the measured intensity ranged from 0 to 255 (low to high).

### 4.8. Immunohistochemistry

Liver tissue sections (5 μm) were prepared for immunohistochemical analysis. After hydration, slides were treated with a peroxidase blocker for 7 min, washed, and followed by blocking with serum-free protein block (Agilent, Santa Clara, CA, USA) for 1 h. Primary antibodies (diluted 1:100) were applied to the slides and incubated overnight at 4 °C. After washing, slides were incubated with streptavidin-conjugated secondary antibody (Dako™ Real Envision, Agilent) for 30 min, followed by incubation with 3,3′-diaminobenzidine for colorization.

### 4.9. Statistical Analysis

Statistical analyses were performed using Prism 10.2.3 (GraphPad, La Jolla, CA, USA). Mean ± standard deviation represented the observed values. Differences among groups were evaluated by analysis of variance, followed by Tukey’s multiple comparison tests for pairwise comparisons. The significance level was set at *p* < 0.05.

## 5. Conclusions

We demonstrated that the administration of 4HR increases glucose uptake in a dose-dependent manner. In cell culture experiments, treatment with 4HR led to elevated expression levels of Glut4, p-AMPK, and Ac-H3. In STZ-induced animal models, 4HR administration resulted in a decrease in blood glucose levels, an effect comparable to insulin administration. Additionally, 4HR increased the levels of Glut4, p-AMPK, and Ac-H3 in the liver. Periodic injections of 4HR also mitigated the weight loss observed in STZ-injected animals. These findings suggest that 4HR administration reduces blood glucose levels in STZ-injected animals through a pathway involving Glut4, p-AMPK, and Ac-H3.

## Figures and Tables

**Table 1:** The summary of used antibodies.

Name	CAT#	Manufacturer	Dilution Ratio	
			WB	IHC
Glut1	sc-377228	Santa Cruz Biotech. Santa Cruz, CA, USA	1:1000	-
Glut4	sc-53566	Santa Cruz Biotech. Santa Cruz, CA, USA	1:1000	1:100
AMPKα1/2	sc-74461	Santa Cruz Biotech. Santa Cruz, CA, USA	1:1000	-
Ac-H3	sc-56616	Santa Cruz Biotech. Santa Cruz, CA, USA	1:1000	1:100
β-actin	sc-47778	Santa Cruz Biotech. Santa Cruz, CA, USA	1:1000	-
p-AMPK	07-681	Merck, Rahway, NJ, USA	1:1000	1:100

(CAT#: catalogue number, WB: Western blot, IHC: immunohistochemistry).

## Data Availability

The original contributions presented in the study are included in the article, further inquiries can be directed to the corresponding authors.
